# A comparative analysis of dendrometric, macromorphological, and micromorphological characteristics of *Pistacia atlantica* subsp. *atlantica* and *Pistacia terebinthus* in the middle Atlas region of Morocco

**DOI:** 10.1515/biol-2022-0941

**Published:** 2024-09-17

**Authors:** Mohammed Bassouya, Mohamed Chedadi, Jawhari Fatima Zahra, Mohammed Kara, Amine Assouguem, Riaz Ullah, Mohamed A. Ibrahim, Ahmed Bari, Hafize Fidan, Lafraxo Soufyane, Abdellatif Alami, Amina Bari

**Affiliations:** Laboratory of Biotechnology, Environment Agrifood and Health, Faculty of Sciences Dhar El Mahraz, Sidi Mohamed Ben Abdellah University, Fez, Morocco; Laboratory of Biotechnology, Conservation and Valorisation of Naturals Resources (LBCVNR), Faculty of Sciences Dhar El Mehraz, Sidi Mohamed Ben Abdellah University, B.P. 1796 Atlas, Fez, 30000, Morocco; National School of Agriculture of Meknes, Department of Plant Protection and Environment, Meknes, Morocco; Laboratory of Functional Ecology and Environment, Faculty of Sciences and Technology, Sidi Mohamed Ben Abdellah University, Imouzzer Street, P.O. Box 2202, Fez, Morocco; Department of Pharmacognosy, College of Pharmacy, King Saud University, Riyadh, 11451, Saudi Arabia; Department of Pharmaceutics, College of Pharmacy, King Saud University, Riyadh, 11451, Saudi Arabia; Department of Pharmaceutical Chemistry, College of Pharmacy, King Saud University, Riyadh, 11451, Saudi Arabia; Department of Tourism and Culinary Management, Faculty of Economics, University of Food Technologies, 4000, Plovdiv, Bulgaria; Laboratory of Applied Organic Chemistry, Faculty of Sciences and Technology of Fez, Sidi Mohamed Ben Abdellah University, Route d’Immouzer, Fez, Morocco

**Keywords:** *Pistacia atlantica* subsp. *atlantica*, *Pistacia terebintus*, morphology, stomatal density, scanning electron microscopy

## Abstract

The genus *Pistacia*, with its species having notable ecological, economic, and medicinal implications, demonstrates remarkable environmental adaptability. The central objective of the study is to analyze interspecific variations between *Pistacia atlantica* subsp. *atlantica* and *Pistacia terebinthus* across three distinct bioclimatic zones in the Middle Atlas region of Morocco. The methodology includes collecting dendrometric measurements and conducting macromorphological examinations on these two taxa, with a detailed analysis of 27 qualitative and quantitative variables. A micro-morphological analysis of leaves, using scanning electron microscopy (SEM), is employed to explore specific features such as size and stomatal density, as well as qualitative aspects like epidermal cell shape and trichomes. Dendrometric measurements have revealed that the canopy surface and the number of trunks per tree can serve as distinctive features between the two species. Regarding the sex ratio of *Pistacia atlantica* subsp. *atlantica*, 59% of the examined trees are males, primarily associated with the jujube tree in arid zones and the dwarf palm in humid areas. In contrast, female *Pistacia terebinthus* exhibit a similar percentage, predominantly associated with oak groves and cade juniper in their distribution areas. Principal component analysis of biometric measurements emphasized a significant disparity between the two species, representing 60.25% of the total variance. The use of SEM unveiled new features facilitating the identification of the two species. By leveraging the macromorphological and micromorphological variability of pistachio trees, we can qualify those best suited to diverse bioclimates. In this regard, we suggest incorporating them into reforestation and rehabilitation programs aimed at restoring our declining ecosystems.

## 1 Introduction

The Mediterranean flora in general, and the Moroccan flora in particular, is now much better understood than it was 20 years ago. Systematic research, whether of a traditional or modern (molecular) nature, has refined our knowledge of various taxa (families, genera, or species) [1]. Our research delves into the present condition of iconic Moroccan ecosystems, with a specific focus on two members of the Anacardiaceae: PA (*Pistacia atlantica* subsp. *atlantica*) and PT (*Pistacia terebinthus*).

With around ten species of pistachio trees, some of them share similar physical characteristics, posing challenges in accurate identification when observed in their natural habitats. This challenge is particularly evident for the PA and PT, given their morphological resemblances. Overcoming this potential confusion and ensuring precise identification necessitates a comprehensive study of these species [2].

The genus *Pistacia* comprises ten species characterized by alternate, pinnate-compound leaves [3]. Also, from a taxonomic perspective, establishing the limits of Anacardiaceae has been challenging; that is to say, not long ago, Anacardiaceae and Bursuaceae were grouped under the family Terebinthaceae [4]. According to Zohary [5], PA has been classified into four distinct eco-geographical varieties: cabulica, latifolia, kurdica, and finally, atlantica, originating from the Maghreb region [6], while Álvarez et al. [7] have elevated the first three varieties to the status of subspecies; moreover, other authors considered some of them as separate species [8–11].

However, it is worth noting that the classification proposed by Rechinger [12] was not accepted by Iranian botanists [13]. PA is naturally found from the Mediterranean basin to Central Asia. It is a dominant tree in its natural spaces and plays a significant economic role in these regions. The leaves are imparipinnate with 5–9 lanceolate leaflets, measuring 26–70 mm in length and 8–22 mm in width. The petioles are winged and are extra-axillary, ranging from 7 to 15 cm in length [14].

PT is a shrub or small tree and typically range in height from 2 to 6 m, occasionally reaching up to 12 m. Their deciduous leaves, either impari- or paripinnate, measure between 10 and 19 cm in length and 6–19 cm in width, eventually becoming leathery. Petioles are usually rounded, rarely flattened, and the rachis lacks winged extensions. Leaflets, numbering from 3 to 11, are typically 3.5–8 cm long and 1–3.1 cm wide, with an average ratio of about 2.7:1. They are arranged opposite to subopposite and vary from ovate to narrowly ovate, often with a pointed tip and a fine pubescence or smooth surface. The terminal leaflet, if present, tends to be smaller than the lateral ones, measuring between 1 and 6 cm long and 5 and 20 mm wide. Staminate panicles, reaching up to 8 cm long, feature diffuse branching and large bracts with long white trichomes. Pistillate panicles, up to 15–23 cm long, become more robust in fruit and may have a slightly hairy or smooth surface. Initially reddish, the drupes eventually ripen to a blue color, completing the characteristic features of these plants [15].

PT is native to Eastern Mediterranean countries, where it is commonly found on rocky sites with open vegetation. It avoids the driest and coldest locations [16].

Morphological characterization of a plant represents a critical and significant trait as it encompasses growth and developmental profile while also playing a role in formulating conservation strategies. When distinguishing between taxa or for taxonomy purposes, it is essential to consider the various levels and degrees of intraspecific and interspecific variations [17].

Indeed, these two taxa are characterized by their intraspecific diversity, broad geographic distributions, and therapeutic uses by different ethnic groups in Asia and North Africa [18].

On the other hand, our comparison also encompasses the phytodermic and stomatal characteristics of the two taxa under study to explore potential variations between them. It should be pointed out that the morphology, distribution, and behavior of stomata exhibit responsiveness to a diverse range of signals, ranging from intracellular signaling to global climate changes [19].

Precise identification of the two taxa is essential for establishing a combined strategy for conservation and improvement. Indeed, species identification involves three distinct types of characterization: morphological, biochemical, and molecular. In this study, we specifically focused on the first type, directing our attention to macromorphological and micromorphological characterization, which form the fundamental basis for species classification.

## 2 Materials and methods

### 2.1 Sampling area

**Figure 1:**
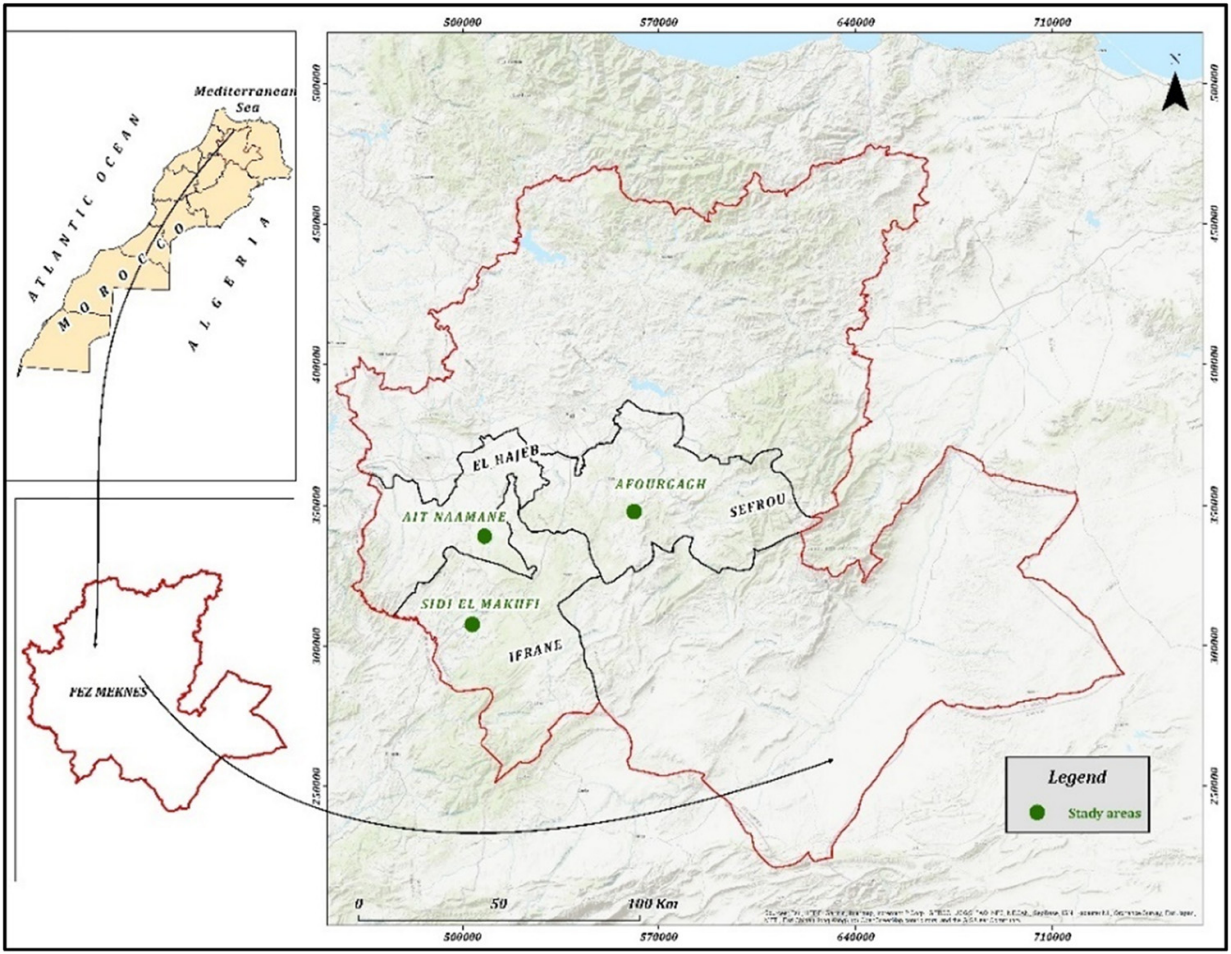
Map of the study area and sampling stations (ARC-GIS program).

Trees of PA and PT used in this study were sampled from three regions (Figure 1) located in different bioclimatic zones of the Middle Atlas in Morocco (Table 1). It is important to emphasize that the populations of PA and PT are scattered or isolated. Therefore, we sampled our trees at various locations in the same study area based on the presence of both species. Regarding PT, we noticed that this taxon is distributed in humid areas, with a few isolated individuals found in the early stages of the semi-arid climate, particularly in our Amghas zone. In other words, as aridity increases, PT disappears regardless of altitude.

**Table 1:** Summary of the main climatic characteristics of the studied stations [23–25]

Study areas		Latitude	Longitude	Altitude	Substrate type	Bioclimatic stage
Ait Naamane	*Pistacia atlantica* Desf.	33°41'56.1″N	5°21'23.2″W	1,023	Shallow red soil resting on Lias limestones and dolomites	Sub-humid, ranging from cool to cold
	*Pistacia Terebinthus* L.	33°41'50.9″N	5°21'25.1″W	1,020		
Lac Afourgagh	*Pistacia atlantica* Desf.	33°36'45.9″N	4°53'02.6″W	1,449	Shallow red soil resting on a carbonate series, typically dolomitic	Semi-arid with cool winters
	*Pistacia Terebinthus* L.	33°36'57.7″N	4°52'57.1″W	1,425		
Amghas	*Pistacia atlantica* Desf.	33°68'63.9″N	5°37'01.1″W	1,043	Schistose-sandstone and quartzitic deposits	Semi-arid with a temperate winter
	*Pistacia Terebinthus* L.	33°37'12.5″N	5°25'45.2″W	1,047		

## 3 Dendrometric characteristics of trees

Due to the uneven distribution of trees, an initial visual count was conducted on each slope of every station. Subsequently, various parameters were assessed, including plant sex, circumference, height, canopy, associated flora, number of trunks, and tree vigor. The selection of trees was made randomly and based on the presence of the two studied taxa. We chose only trees that had reached a flowering stage in order to know the sex of each tree (Table 2).

**Table 2:** Dendrometric characteristics measured for trees in distinct populations

	Circumference PA (cm)	Height PA (cm)	Surface of canopy PA (cm)	Circumference PT (cm)	Height PT (cm)	Surface of canopy PT (cm)
Amghas	204.5a ± 75.3 (92–335); 36.82	823.4b ± 414.1 (131–1,550); 50.30	3,821c ± 1,298 (1,620–6,468); 33.98	72d ± 23.75 (50–105); 32.98	573.3e ± 65.55 (505–670); 11.43	2,010f** ± 393.2 (16.33–2,575); 19.57
Ait naamane	299.3a ± 113.9 (60–365); 38.07	1399b ± 398.5 (710–1,860); 28.5	3,719c ± 1,068 (2,010–5,401); 28.71	158.5d ± 79.36 (60–340); 50.06	936.9e ± 207.3 (705–1,420); 22.13	3,042f** ± 840.8 (2,010–5,401); 27.64
Afourgagh	258.9a ± 95.16 (102–380); 36.75	1234b ± 486.4 (720–2,270); 39.4	5,483c* ± 1,390 (3,580–7,850); 25.35	191.4d ± 108.2 (70–312); 56.5	1,181e ± 155.5 (955–1,365); 13.16	4,816f*** ± 1,965 (3,203–8,236); 40.8

Letters a–f represent the correlation coefficient of variables measured with the station, according to Tukey’s multiple comparisons test (*p* < 0.05). Values identified by the same letter show no significant difference. *p < 0.05; **p < 0.01; ***p < 0.001. Mean Avg; Standard deviation: SD; Range: Min-Max; CV: coefficient of variation (%); PA: Atlas pistachio; PT: Terebinth pistachio.

Height was measured using a geometric principle based on the Bucher’s cross, while circumference was measured with a tape measured at 1.30 m from the ground [20].

Although the exact calculation of the crown area is theoretically impossible [21], it can be approximated by utilizing directly measurable values such as the crown height and diameter using the following formula:

\[S={[}\pi (d1\cdot d2)]/4,]\]

where *S* represents the crown area at the ground level in square meters, *d*1 is the ground diameter of the north–south crown, and *d*2 is the east–west crown diameter at the ground level [22].

Statistical analysis of data: to assess the consistency or disparity of the collected dendrometric parameters, various statistical indicators were utilized. The standard deviation (*σ*) and coefficient of variation (CV) were calculated among these parameters, providing insights into the dispersion and distribution of individuals around the mean. Furthermore, a two-factor analysis of variance was applied, accompanied by the use of Tukey’s test for both variance analysis and mean comparison.

## 4 Morphological analysis

### 4.1 Sampling of the plant material

Due to the scattered distribution of individuals of the studied species, sample collection was carried out subjectively by selecting the most accessible subjects in the field. Consequently, 30 trees were measured in each of the three study stations. Given the deciduous nature of PA, periodic field trips were conducted throughout the year 2022 to collect leaves, inflorescences, and fruits at different stages of development. Morphological characteristics were chosen as the main analysis parameters, as they are frequently used by taxonomists to differentiate between taxa. These characteristics provide crucial information about the variability of adaptations and are essential for a better understanding of natural population differentiation, as indicated in ref. [22].

### 4.2 Measured parameters

**Figure 2:**
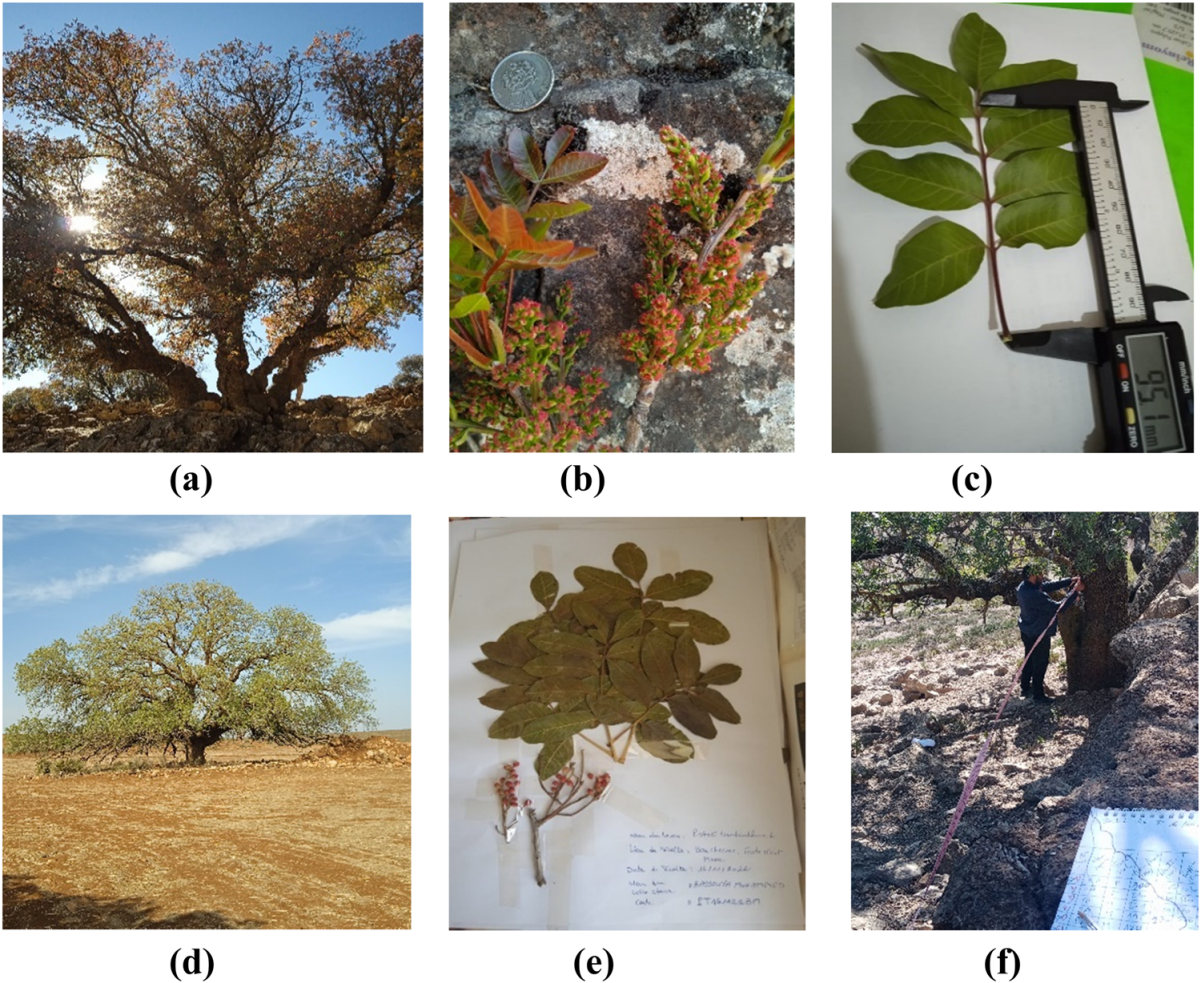
Plant material used and certain measurement techniques: (a) Tree of Pistacia terebinthus. L (PT); (b) inflorescence of PT; (c) leaf diameter measurements using a caliper; (d) tree of *Pistacia atlantica* subsp. *atlantica* (PA); (e) Herbarium; and (f) dendrometric measurements.

The reference samples for each institution were sorted at the Herbarium of the Dher el Mehrez Faculty of Science, specifically the “LBEAS” laboratory (Figure 2).

At each location, a random selection of 20 trees was made. Subsequently, each plant was sampled, including 20 leaves and 20 fruits. The leaves and fruits were naturally dried and stored in the laboratory. Biometric and morphological measurements were conducted following the method described by the descriptors of the pistachio tree [26].

In total, 27 morphological characteristics were measured, consisting of 13 qualitative traits and 14 quantitative traits. The qualitative traits are represented in Table 3, while the quantitative traits are presented in Table 4.

**Table 3:** Character encoding of qualitative morphological traits

Character	Code	Value
Size of the terminal leaflet	SiTL	1; resembling the basal leaflets 2; greater in size than the basal leaflets3; lesser in size than the basal leaflets
Shape of the terminal leaflet	ShTL	1; lanceolate widened 2; elliptical 3; oval 4; round oval 5; rounded 99; other terminal leaflet apex shape
Shape of terminal leaflet apex	ShTLa	1; pointed or tapering 2; sharp tip 3;slightly sharp tip 4;rounded 5;Indented 6; other shapes
Shape of terminal leaflet base	ShTLb	1; attenuate 2; rounded 3; truncate 4; inclined 99; other
Margin of terminal leaflet	MTL	1; flat 2; wavy
Shape of the petiole	ShP	1; flattened 2; rounded 3; rounded with a straight upper surface
Leaf color	LC	1; light green 2; green 3; deep green
Unduly from the central leaf vein	UCLV	1; glabrous 2; sparsely pubescent
Shape of flowering buds	ShFB	1; broad oval 2; narrow oval 3; conical
Color of the flowering buds	CFB	1; burnished 2; Blonde 3; brown 4; Russet
Abundance of inflorescence	AIn	3; sparse 5; moderate 7; dense
Shape of the fruit	ShFr	1; round (*l*/*w* < 1.5) 2; ovoid (1.5 < *l*/*w* < 1.8) 3; elongated (*l*/*w* > 1.8) 4; narrowed cordiform 5; cordiform
Apex of the shell	AS	1; flattened 2; rounded 3; symmetrical treble
		4; asymmetrical treble
Cell shape	Csh	1; irregular 2; isodiametric 3; polygonal
Type of stomata	Ts	1; anomocytic 2; actinocytic 3; paracytic
Type of trichome	Tt	1; Absent 2; glandular 3; uncinate/hooked

**Table 4:** Characteristics of the quantitative traits measured for the leaves, inflorescences, and fruits of the three populations

Characteristics	Avg ± SD; Extent					
	Ait Naamane «Area: 1»		Lac Afourgagh «Area: 2»		Amghas «Area: 3»	
	PA	PT	PA	PT	PA	PT
Leaf length (cm)	15.81 ± 2.16a (11.8–19.8)	15.27 ± 2.79a (10.8–19.8)	11.15 ± 1.98a (8.3–15.6)	12.05 ± 3.49a (5.5–19)	13.22 ± 2.84a (7.2–17.1)	14.19 ± 3.18a (8.7–19.9)
Leaf width (cm)	11.71 ± 1.54a (9.6–15.4)	12.44 ± 3.06a (7.33–17.71)	8.46 ± 1.78a** (5.4–11)	10.6 ± 2.39 b** (7.1–15.2)	11.79 ± 2.58a*** (6.2–15.3)	11.81 ± 1.91 b*** (8.9–15.9)
Number of leaflets	10.95 ± 2.04a*** (6–13)	8.5 ± 1.43b*** (6–11)	8.15 ± 2.13a (4–11)	8.75 ± 1.95a (5–12)	6.6 ± 1.60a (3–9)	7.15 ± 1.79a (3–11)
Length of the terminal leaflet (cm)	4.35 ± 2.02a (0–6.7)	4.57 ± 2.08a (0–8.09)	3.16 ± 1.50a (0–4.5)	3.45 ± 1.21a (1.1–6)	4.23 ± 2.49a (0–7.4)	3.39 ± 2.53a (0–8.5)
Width of the terminal leaflet (cm)	0.935 ± 0.45a*** (0–1.4)	1.69 ± 0.83b*** (0–3.07)	1.07 ± 0.50a** (0–1.5)	1.69 ± 0.63b** (0.5–2.9)	1.38 ± 0.80a (0–2.3)	1.75 ± 1.29a (0–4.5)
Length/width ratio	4.109 ± 2.09a** (0–8)	2.52 ± 1.0b** (0–3.92)	2.52 ± 1.14a (0–3.5)	2.09 ± 0.37a (1.5–3.0)	2.49 ± 1.34a* (0–3.82)	1.55 ± 1.08b* (0–3.25)
Fruit length (mm)	7.99 ± 0.89a*** (6.2–9.6)	4.34 ± 0.42b*** (3.8–5.2)	7.68 ± 1.06a*** (5.9–9.2)	6.04 ± 0.55b*** (4.9–7.2)	7.9 ± 0.62a*** (6.4–9)	6.64 ± 0.35b*** (5.8–7.2)
Fruit width (mm)	5.63 ± 0.88a*** (4.1–6.8)	3.32 ± 0.25b*** (3–3.9)	6.04 ± 0.8a*** (4.8–7.8)	4.9 ± 0.66b*** (3.4–6.1)	6.05 ± 0.66a* (5.2–7.9)	5.67 ± 0.37b* (5.1–6.4)
Fruit thickness (mm)	5.545 ± 0.84a*** (4.1–6.7)	2.12 ± 2.89b*** (1.5–2.7)	5.90 ± 0.86a*** (5–7.6)	4.84 ± 0.60b*** (3.7–6)	4.33 ± 0.39a*** (3.7–5.2)	5.41 ± 0.40b*** (5–6.4)
Inflorescence rachis length (cm)	4.79 ± 1.93a*** (1.3–7.6)	8.83 ± 0.1b*** (6.8–10.8)	10.90 ± 1.21a*** (9–14.2)	13.26 ± 2.59b*** (8.7–17.2)	12.4 ± 2.06a* (8.5–15.8)	14.64 ± 4.32b* (7.5–23.1)
Number of primary lateral inflorescence	15.1 ± 0.45a (14–16)	16.65 ± 3.91a (7–24)	16.20 ± 2.75a*** (12–21)	12.3 ± 2.83b*** (7–18)	15.6 ± 3.05a (10–21)	14.2 ± 3.40a (8–20)
Almonds length (mm)	5.3 ± 0.3a (4.8–5.8)	5.453 ± 0.60a (4.2–6.7)	5.36 ± 0.27a*** (4.9–.5.8)	4.43 ± 0.76b*** (2.9–5.5)	5.19 ± 0.39a (4.2–5.8)	5.03 ± 0.35a (4.4–5.8)
Almonds width (mm)	5.0 ± 0.32a (4–5.3)	4.94 ± 0.52a (4.3–6.2)	5.06 ± 0.40a (4.1–5.8)	4.04 ± 0.98b*** (1.8–5.2)	4.33 ± 0.37a (3.5–5)	4.29 ± 0.30a (3.8–4.8)
Almonds thickness (mm)	4.88 ± 0.34a (4–5.3)	4.97 ± 0.45a (4.4–6.2)	4.13 ± 0.31a (3.4–4.6)	4 ± 0.91a (1.9–5.1)	3.89 ± 0.22a* (3.4–4.2)	3.66 ± 0.31b* (3.1–4.1)

Mean: Avg; Standard deviation: SD; Range: Min–Max; values marked with the same letters (a;b) are not significantly different. *p < 0.05; **p < 0.01; ***p < 0.001.

## 5 Micromorphological characteristics of leaflets

### 5.1 Observations under a photon microscope

For optical microscopy, observations were conducted on epidermal impressions obtained using the epidermal sampling method. The technique involves applying a sufficiently thick layer of transparent nail polish to both sides of a fresh leaf. Once the nail polish has completely dried in ambient air, the peel of the nail polish, reproducing the topography of the leaf, is gently removed with tweezers and spread onto a slide previously moistened with a drop of water. Observations were made at magnifications of ×10 and ×40. The slides are stored in the Laboratory of Biotechnology, Environment, Agriculture, and Health at Sidi Mohamed Ben Abdellah University.

### 5.2 Scanning electron microscopy (SEM) observations

The sample preparation method for SEM observation depends on the information sought, including the topography, microstructure, nature of the sample, and its presentation form, such as biological samples or powders [27].

The epidermal surfaces of the leaves were examined using a scanning electron microscope. Three specimens were taken from each site, each comprising a 5 mm^2^ section of the dry leaf surface (abaxial and adaxial surfaces). SEM images were digitally recorded at various levels of magnification with a JSM-IT500HR at the Regional University Interface Center (CURI) of Sidi Mohamed Ben Abdellah University in Fès. Observations and measurements focused on ten qualitative and quantitative characteristics of trichomes and stomata. Qualitative features included the shape of the epidermal cells, stomatal shape, stomatal complex type, and trichome type. Quantitative features encompassed stomatal density on the upper and lower surfaces, as well as the length and width of stomata.

## 6 Results

### 6.1 Dendrometric characteristics

#### 6.1.1 Quantitative measurements

Comparison of the conformation of samples from PA and PT trees. Sixty trees were studied in three selected zones, with 20 trees per zone.

According to our dendrometric measurements summarized in Table 3, a significant phenotypic difference was observed in the field between PA and PT, specifically in terms of canopy surface and the number of trunks per tree. For each species, there was no significant difference in the tree height and circumference between the three zones. Indeed, the results for height, circumference, and canopy surface were consistently higher (1,399 cm, 299.3 cm, 5,483 cm^2^) for PA than for PT in all three zones.

**Figure 3:**
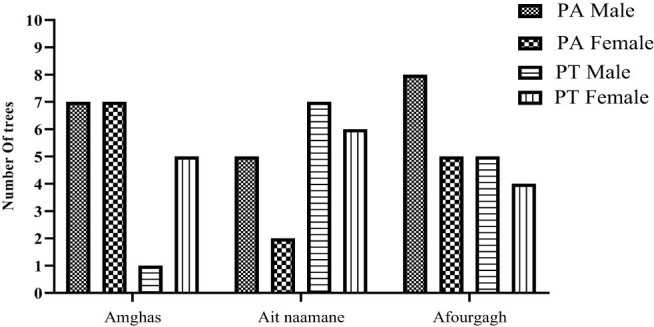
Number of male and female individuals in the three zones; PA: *Pistacia atlantica Desf* and PT: *Pistacia terebinthus.*

For PA, 59% of the examined trees were male, and the identical percentage was noted for female trees of PT (Figure 3). It is crucial to underscore that no instances of hermaphroditism were detected in any of the populations under study.

### 6.2 Qualitative measurements

**Figure 4:**
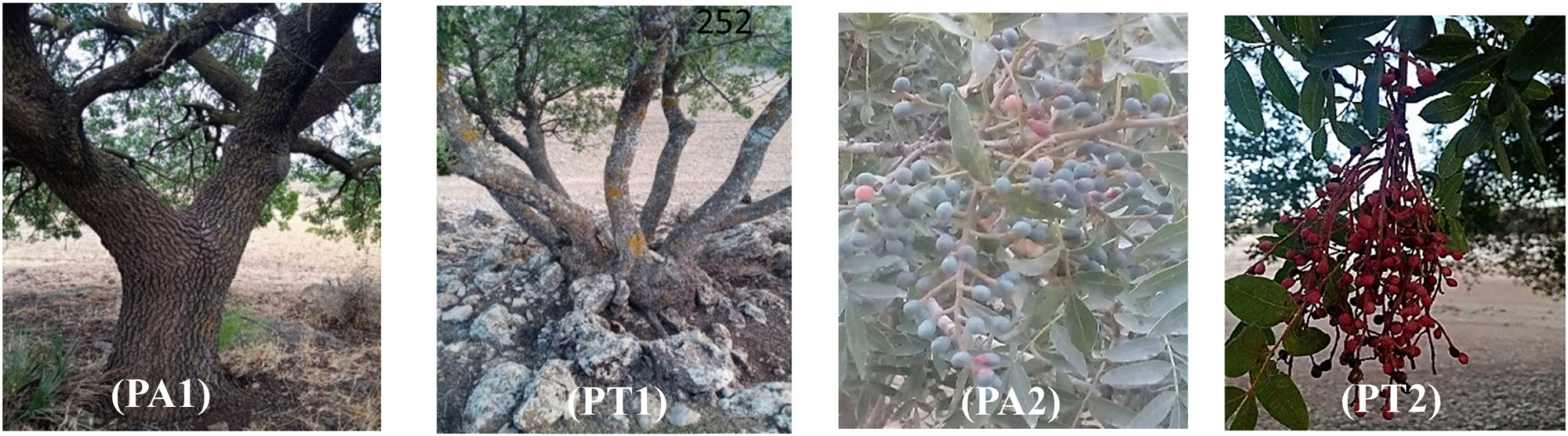
PA1: individualized trunk in PA; PT1: branching trunk in PT; PA2: ripe fruits of PA; and PT2: ripe fruits of PT.

As regards the tree trunks of the two taxa studied, we found that PA trees are mainly individualized, with rare cases of two trunks (Figure 4).

With regard to the vegetation associated with PA, the analysis highlights the presence mainly of *Ziziphus lotus* L. (Family: Rhamnaceae) at Amghas, as well as *Chamaerops humilis* L. (Arecaceae) and *Quercus ilex* L. (Fagaceae) at Afourgagh and Ait Naamane. PT, on the other hand, is mainly accompanied by *Quercus ilex* L. (Fagaceae), *Pistacia lentiscus* L. (Anacardiaceae), and *Juniperus oxycedrus* L. (Cupressaceae) in the three zones studied.

**Figure 5:**
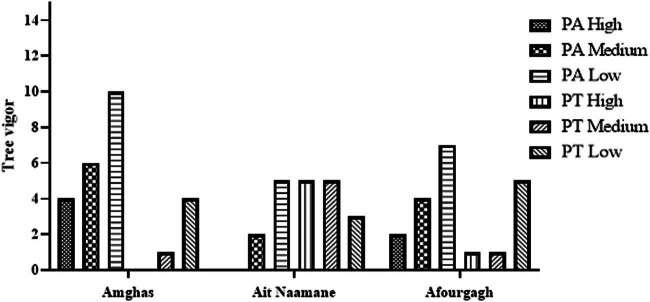
Number of plants according to vigor.

Regarding tree vigor, 50% of the trees of both species showed low vigor, while only 20% of the specimens represented vigorous trees (Figure 5). In terms of trunk, all measured PT trees have branching (not-individualized) trunks, ranging from 2 to 7 stems in some cases.

## 7 Morphological traits of leaves, inflorescence, and fruits

### 7.1 Inter-species variability

The leaves of PA observed in different locations showed an average length of 13.39 ± 2.32 cm, varying from a minimum of 7.2 ± 2.84 cm to a maximum of 19.8 ± 2.16 cm. Leaf width was between 5.4 ± 1.78 cm and 15.4 ± 1.54 cm, with an average width of 10.65 ± 1.96 cm.

For leaves of PT, length ranged from 5.5 ± 3.49 cm to 19.9 ± 3.18 cm, with an average length of 13.83 ± 3.15 cm. The calculated width had an average variation of 11.61 ± 2.45 cm, ranging from a minimum of 7.1 ± 2.39 cm to a maximum of 17.71 ± 3.06 cm (Table 4).

**Figure 6:**
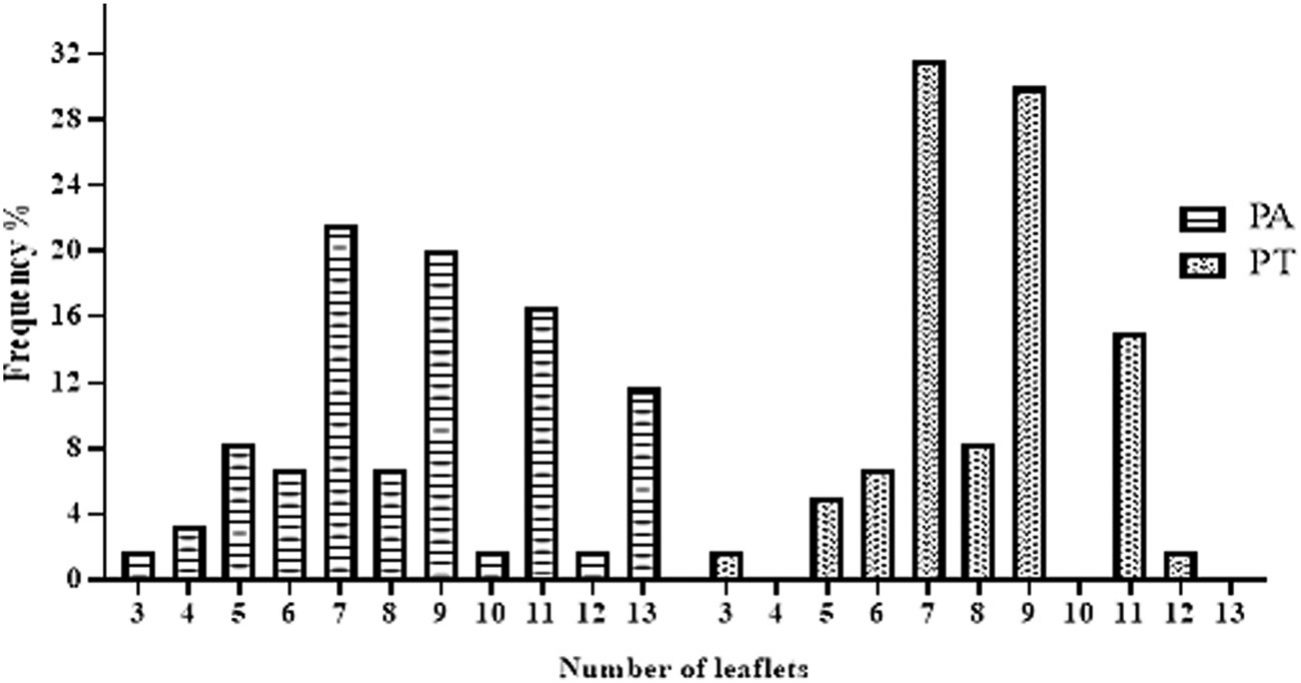
Frequencies of the number of terminal leaflets.

With regard to leaf morphology, both species are mainly characterized by an odd-pinnate arrangement of their leaflets. In the case of PA, the average number of leaflets was 8.56 ± 1.92. The most frequently observed number of leaflets was 7, representing 21.6% of the cases. In contrast, PT had an average of 8.13 ± 1.72 leaflets per leaf. The number of leaflets most frequently encountered was 7, with a frequency of 31.6% (Figure 6).

**Figure 7:**
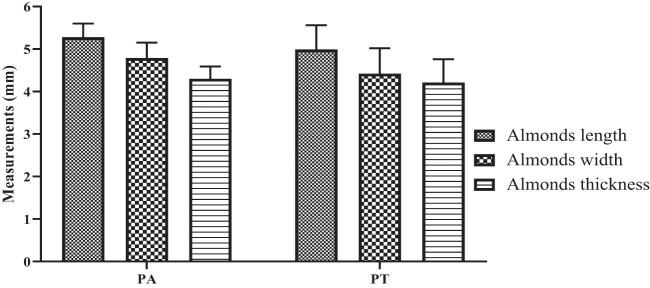
Fruit size measurements.

The comparison of the ratios between the length and width of the terminal leaflet in the PA and the PT within three distinct stations reveals significant differences. The average length/width ratio for PA was 3.03 + 1.32, which was significantly higher than that of the PT estimated to be 2.05 + 0.82. The analysis of the dimensions of the fruits (Figure 4) reveals a marked distinction between PA and PT. The seeds of PA had higher values, with an average of 7.99 ± 0.89 for the length, while width and thickness were 6.05 ± 0.66 and 5.90 ± 0.86, respectively. For PT, the average width, length, and thickness were 6.64 ± 0.35, 5.67 ± 0.37, and 5.41 ± 0.40, respectively (Figure 7).

**Figure 8:**
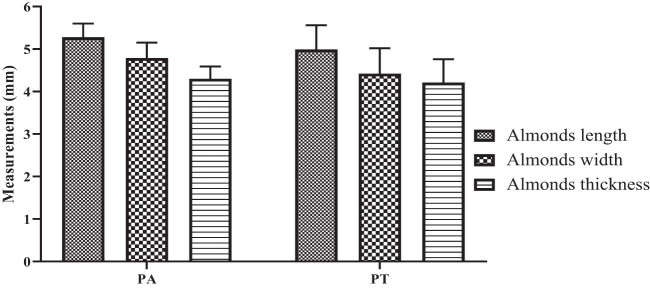
Almond size measurements.

The length, width, and thickness of the almond of PA generally were greater than those of PT. The analysis of the dimensions of the almonds reveals a marked distinction between the two species examined. For PA, the length, width, and thickness of the almond were 5.283 ± 0.96, 4.79 ± 0.363, and 4.3 ± 0.29, respectively; and for PT, they were 4.996 ± 0.57, 4.42 ± 0.6, and 4.2 ± 0.556, respectively (Figure 8).

Morphometric analysis also revealed a significant difference in the length of the inflorescence rachis between the PT and PA. Indeed, the length of the rachis of the inflorescence is greater in PT than in PA. Also, PA has a higher number of primary lateral inflorescential branches compared to PT (Table 4).

## 8 Inter-population variability

### 8.1 Principal component analysis (PCA)

**Figure 9:**
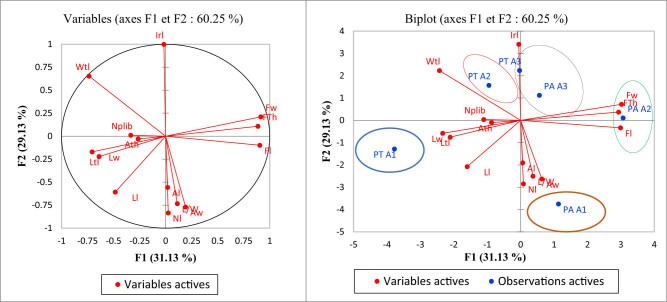
Correlation circle and individual projection onto factors 1 and 2.

The 14 quantitative morphological characters of specimens from various populations of PA and PT were subjected to PCA. The main emphasis of the interpretation of the results was placed on the first two axes, namely axis 1 and axis 2. The information provided by these two selected axes represents 60.25% of the total variance (Figure 9). To identify the variables responsible for the variations along the selected axes, we used the correlation between the variables and the factors (Table 5). Only the first two factors (axes) present variables with factor weights ≥0.6. It is obvious that most of the measured variables are strongly negatively correlated with axis 1 and axis 2. However, the width of the terminal leaflet is negatively correlated with axis 2, while the length of the inflorescence rachis is positively correlated with axis 2. On the other hand, the parameters of the fruits are strongly correlated with axis 1. It is clearly observable that the populations of PA and PT are distinguishable, which indicates morphological differences in the characteristics of the leaves, flowers, and fruits. The traits (variables) responsible for this distinction are those that have high correlations with the axes concerned (≥0.6).

**Table 5:** Correlations between variables and factors: correlations ≥ 0.6 are in bold

	F1	F2	F3	F4	F5
Leaf length	−0.484	**−0.608**	−0.507	−0.361	−0.091
Leaf width	**−0.706**	−0.170	−0.394	−0.448	−0.341
Number of leaflets	0.025	**−0.834**	0.264	−0.270	0.400
Length of the terminal leaflet	**−0.639**	−0.221	**0.609**	−0.371	−0.186
Width of the terminal leaflet	**−0.736**	**0.654**	−0.085	−0.072	0.138
Length/width ratio	0.112	**−0.733**	0.574	−0.345	0.010
Fruit length	**0.905**	−0.098	−0.042	−0.278	−0.303
Fruit width	**0.915**	0.211	−0.109	−0.260	−0.198
Fruit thickness	**0.888**	0.108	−0.009	−0.335	0.297
Inflorescence rachis length	−0.017	**0.999**	−0.030	−0.007	0.027
Number of primary lateral inflorescence	−0.334	0.011	**0.859**	0.383	0.060
Almond length	0.019	−0.557	−0.540	0.576	−0.256
Almond width	0.192	**−0.770**	−0.222	0.557	0.105
Almond thickness	−0.263	−0.030	−0.825	−0.233	0.442

## 9 Inter-specific variability of qualitative morphological parameters

### 9.1 Leaf parameters

The size of the terminal leaflet exhibits significant variations. In PA, 73.3% of the terminal leaflets resemble those of basal leaflets, whereas in PT, this proportion is 48.3%. Furthermore, 73.3% of the terminal leaflets in PA have an elongated lanceolate form, whereas in PT, it is 71.67% and they have an oval rounded shape. Concerning the apex of the leaflets, 83.3% of the acuminated forms are observed in PA, while 56.66% of the mucronulate forms are noted in PT (Table 6).

**Table 6:** Frequencies (%) for qualitative traits measured in different populations of *Pistacia atlantica* Desf and *Pistacia terebinthus* L.

Provenance species		Amghas		Afourgagh		Ait Naamane	
		*Pistacia atlantica* Desf	*Pistacia terebinthus* L.	*Pistacia atlantica* Desf	*Pistacia terebinthus* L.	*Pistacia atlantica* Desf	*Pistacia terebinthus* L.
Size of the terminal leaflet (STL)	0	0	0	15	0	20	25
	1	100	0	60	75	60	10
	2	0	15	25	25	10	5
	3	0	85	0	0	10	60
Shape of the terminal leaflet (ShTL)	0	0	0	15	0	20	25
	1	100	0	75	0	45	10
	2	0	0	10	0	30	15
	4	0	80	0	85	0	50
	5	0	20	0	15	0	0
Shape of the terminal leaflet apex (ShTLa)	0	0	0	15	0	20	25
	1	100	25	70	25	80	25
	2	0	0	15	0	0	5
	3	0	75	0	75	0	20
	4	0	0	0	0	0	30
Shape of the terminal leaflet base (ShTLb)	0	0	0	15	0	20	25
	1	100	0	85	85	80	75
	2	0	0	0	15	0	0
	3	0	0	0	0	0	0
	4	0	100	0	0	0	0
Margin of the terminal leaflet (MTL)	0	0	0	15	0	20	25
	1	100	100	85	100	70	75
	2	0	0	0	0	10	0
Shape of the petiole (ShP)	0	0	0	15	0	0	0
	1	0	100	0	80	15	100
	2	20	0	10	0	40	0
	3	80	0	75	20	45	0
Leaf color (LC)	0	0	0	15	0	0	0
	1	5	5	0	0	5	15
	2	70	95	25	10	75	85
	3	25	0	70	90	20	0
Unduly from the central leaf vein (UCLV)	1	100	100	100	80	100	100
	2	0	0	0	20	0	0
Shape of the flowering buds (ShFB)	1	0	85	10	85	100	100
	2	100	15	90	15	0	0
Color of the flowering buds (CFB)	1	100	60	25	100	65	100
	2	0	40	0	0	0	0
	4	0	0	75	0	35	0
Abundance of inflorescence (AIn)	3	0	0	0	85	15	55
	5	100	30	85	15	55	45
	7	0	70	15	0	30	0
Shape of the fruit (ShFr)	1	100	100	100	85	100	15
	2	0	0	0	15	0	85
Apex of the shell (AS)	1	0	100	65	10	10	85
	2	100	0	35	90	90	15

Also, the shape of the basal leaflets differs between the two species. In PA, 88.3% of the basal leaflets are attenuated, whereas in PT, this proportion is 53.3%, with 33% adopting an oblique form. Regarding the edge of terminal leaflets, it is flat in PA (85%) and PT (91.66%).

The petiole exhibits differences. In PA, the petiole is rounded and straight on the upper surface (66.6%), while in PT, it is flattened (93.3%). The leaf color varies, being green and dark green in PA (48 and 38.3%, respectively) and similarly in PT (60 and 30%, respectively). However, the color difference was observed in November, the time when the leaves are about to fall. Young leaves of PA are pale when young, while those of PT are reddish. The central leaf veins are glabrous at 100% for both species (Table 6).

### 9.2 Flower and fruit parameters

In PA, the flower buds have a narrowed oval form, while in PT, 90% have an enlarged oval shape. The flower color is reddish-brown in 63.3% of PA and 86.66% of PT. Inflorescence abundance varies, with 80% moderately abundant inflorescences in PA and 46.67% sparsely abundant in PT. The fruit shape of both species is round (*l*/*w* < 1.5) at 100% for PA and 66.67% for PT. The apex of fruits is rounded in PA (75%) and flattened in PT (65%) (Table 6).

## 10 Comparative analysis of micromorphological characters

### 10.1 Quantitative characteristics measured for stomata

According to Table 7, it is observed that stomatal density values in PA range from 150 to 440 stomata per mm² on the abaxial side, with an average of 280.03 stomata per mm². Conversely, on the adaxial side of the leaf, where the number of stomata is lower, the variation ranges between 5 and 85 stomata per mm², with an average of 28.03 stomata per mm².

**Table 7:** Stomatal density in different study areas

Characteristics	Stomatal density of PA on the abaxial side	Stomatal density of PA on the adaxial side	Stomatal density of PT on the abaxial side	Stomatal density of PT on the adaxial
Afourgagh	265.6ac** ± 43.76; 215–340 (16.48)	19.22ac*** ± 12.25; 5–41 (63.7)	290.6ad*** ± 20.22; 265–315 (6.96)	42.89a*** ± 20.23; 17–74 (47.16)
Amghas	221.8b** ± 43.64; 150–275 (19.68)	8.222b*** ± 3.42; 5–15 (41.59)	186.4be*** ± 25.75; 144–214 (13.81)	84.78bc ± 15.03; 60–102 (17.73)
Ait Naamane	358.7bd ± 51.85; 285–440 (14.46)	56.67bd ± 20.62; 20–85 (36.38)	409.2cf*** ± 48.24; 340–480 (11.79)	121.4ac ± 32.61; 74–165 (26.85)

Mean: Avg; Standard deviation: SD; Range: Min–Max; CV: coefficient of variation (%); values marked with the same letters are not significantly different. *p < 0.05; **p < 0.01; ***p < 0.001.

For PT, stomatal density values vary between 144 and 480 stomata per mm² on the abaxial side, with an average of 295.4 stomata per mm². On the adaxial side of the leaf, the number of stomata varies between 17 and 165 stomata per mm², with an average of 83.02 stomata per mm². It is noteworthy that stomatal density also varies based on the origin of the leaves of the two species, with the highest recorded values in Ait Naamane, followed by Afourgagh, and finally, Amghas.

The measurements of the stomatal size on the adaxial and abaxial sides of the leaves of PA and PT revealed significant variations. In the Afourgagh area, the stomata for PT had an average length of 27.56 µm on the abaxial side and 22.34 µm on the adaxial side. The average width was 19.43 µm on the abaxial side and 10.48 µm on the adaxial side. In Amghas, the stomata of PA has an average length of 22.06 µm on the abaxial side and 18.94 µm on the adaxial side, with average widths of 8.607 and 8.863 µm, respectively. Ait Naamane PA had smaller stomata, with an average length of 14.47 µm on the abaxial side and 12.68 µm on the adaxial side. The average widths were 9.584 and 8.724 µm, respectively.

For PT, in Afrougagh, the average length of the stomata was 16.86 µm on the abaxial side and 21.46 µm on the adaxial side. The average widths were 8.343 and 12.32 µm, respectively, in Amghas, the stomata of PT had an average length of 10.53 µm on the abaxial side and 9.184 µm on the adaxial side, with average widths of 7.942 and 7.634 µm, respectively.

Ait Naamane PT had stomata with an average length of 11.77 µm on the abaxial side and 11 µm on the adaxial side, with average widths of 7.52 and 7.64 µm, respectively. These results highlight the variability in the stomatal size between different zones and the two *Pistacia* species studied (Table 8).

**Table 8:** Size of stomata in the abaxial and adaxial sides

			Ait Naamane	Afourgagh	Amghas
*Pistacia atlantica* Desf	Length of stomata on	Abaxial side (µm)	21.31a** ± 3.14;17. 4–26.35 (14.74)	27.33b** ± 3.73; 20.66–34.14 (13.65)	21.11ad** ± 5.12; 13.1–28.71 (24.25)
		Adaxial face (µm)	22.24a ± 2.85; 18.56–27 (12.81)	23.08a ± 4.45; 16.6–2976 (19.27)	19.05a ± 5.4; 10.2–2.47 (28.36)
	Width of stomata on	Abaxial side (µm)	11.66a*** ± 3.77; 5.89–16.4 (32.36)	19.71bc*** ± 2.64; 15.21–24.55 (13.38)	9.011ad ± 5.22; 2–19.1 (57.89)
		Adaxial face (µm)	11.37a ± 2.02; 9.56–15.52 (17.72)	11.21a ± 3.59; 7.02–17.8 (32.04)	8.89a ± 3.66; 5.17–12.41 (26.6)
*Pistacia terebinthus* L.	Length of stomata on	Abaxial side (µm)	15.87ac*** ± 2.88; 11.66–19.15 (18.16)	15.9ad ± 2.92; 10.49–20.66 (18.36)	23.82be*** ± 2.76; 17.85–28.33 (11.59)
		Adaxial face (µm)	26.42a* ± 3.1; 20.8–30.74 (11.74)	21.81b* ± 4.04; 16.84–29.52 (18.54)	25.06a ± 2.71; 20.7–29.09 (10.8)
	Width of stomata on	Abaxial side (µm)	7.12a ± 1.66; 4.7–9.26 (23.33)	8.15ab*** ± 2.57; 4.18–12.47 (31.56)	17.4ac*** ± 2.22; 14.94–21.34 (12.79)
		Adaxial face (µm)	16.5a* ± 3.63; 12.23–22.66 (22.02)	12.62b ± 2.32; 8.77–16.62 (18.37)	12.85b ± 2.72; 9.46–17.72 (21.2)

Mean: Avg; Standard deviation: SD; Range: Min–Max; CV: coefficient of variation (%); values marked with the same letter are not significantly different. *p < 0.05; **p < 0.01; ***p < 0.001.

### 10.2 Types of trichomes

Figure 9 shows the two main types of trichomes observed in all examined leaves, namely glandular and uncinate. Trichomes are primarily present either on the abaxial or adaxial surfaces, with some cases showing the presence on both surfaces, while in other cases, they are absent. In the present study, trichomes were found on both surfaces of PA leaves in the three study zones: Amghas, Afourgagh, and Ait Naamane. Meanwhile, in PT, trichomes were absent in all samples tested from the three leaf sources. Qualitative results regarding trichomes are presented in Table 9.

**Table 9:** Qualitative variables measured for stomata and trichomes

Provenance species		Amghas		Afourgagh		Ait Naamane	
		*Pistacia atlantica* Desf	Pistacia *terebinthus* L.	*Pistacia atlantica* Desf	*Pistacia terebinthus* L.	*Pistacia atlantica* Desf	*Pistacia terebinthus* L.
Cell shape	1	100	100	100	90	100	100
	2	0	0	0	0	0	0
	3	0	0	0	10	0	0
Type of stomata	1	90	90	70	100	80	100
	2	0	10	20	0	0	0
	3	10	0	10	0	20	0
Type of trichome	1	20	100	0	100	30	100
	2	70	0	20	0	20	0
	3	10	0	80	0	50	0

**Figure 10:**
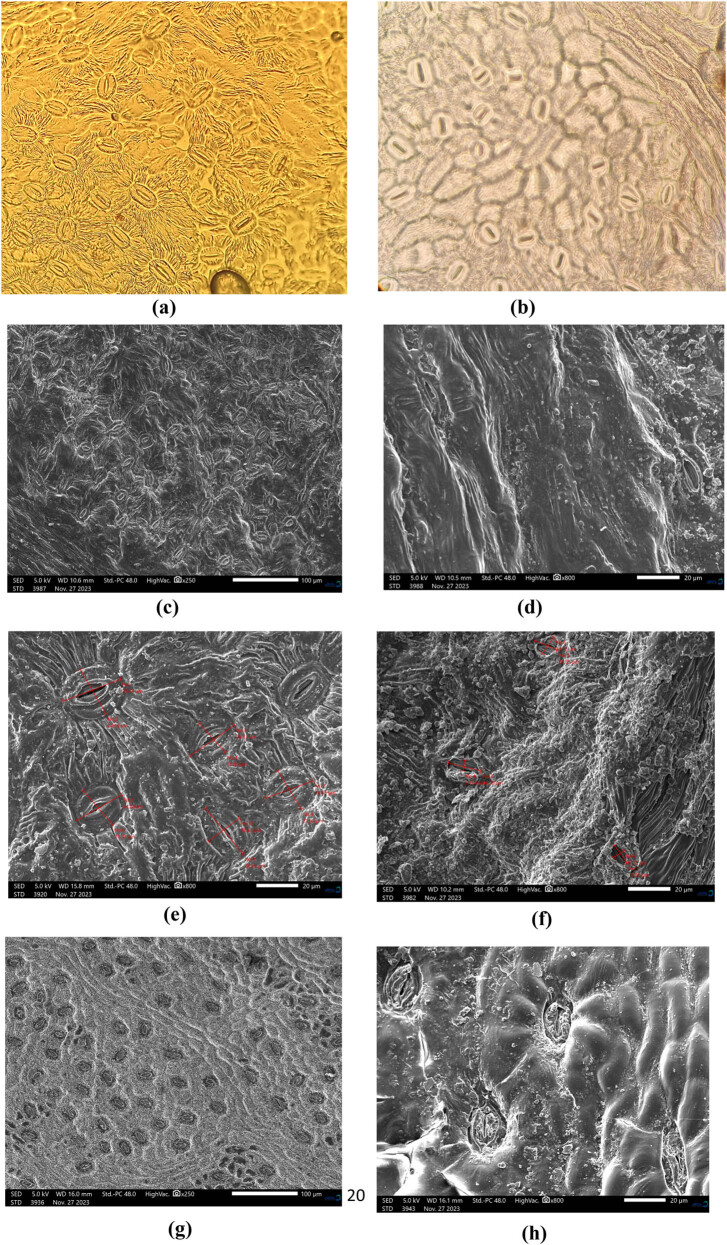

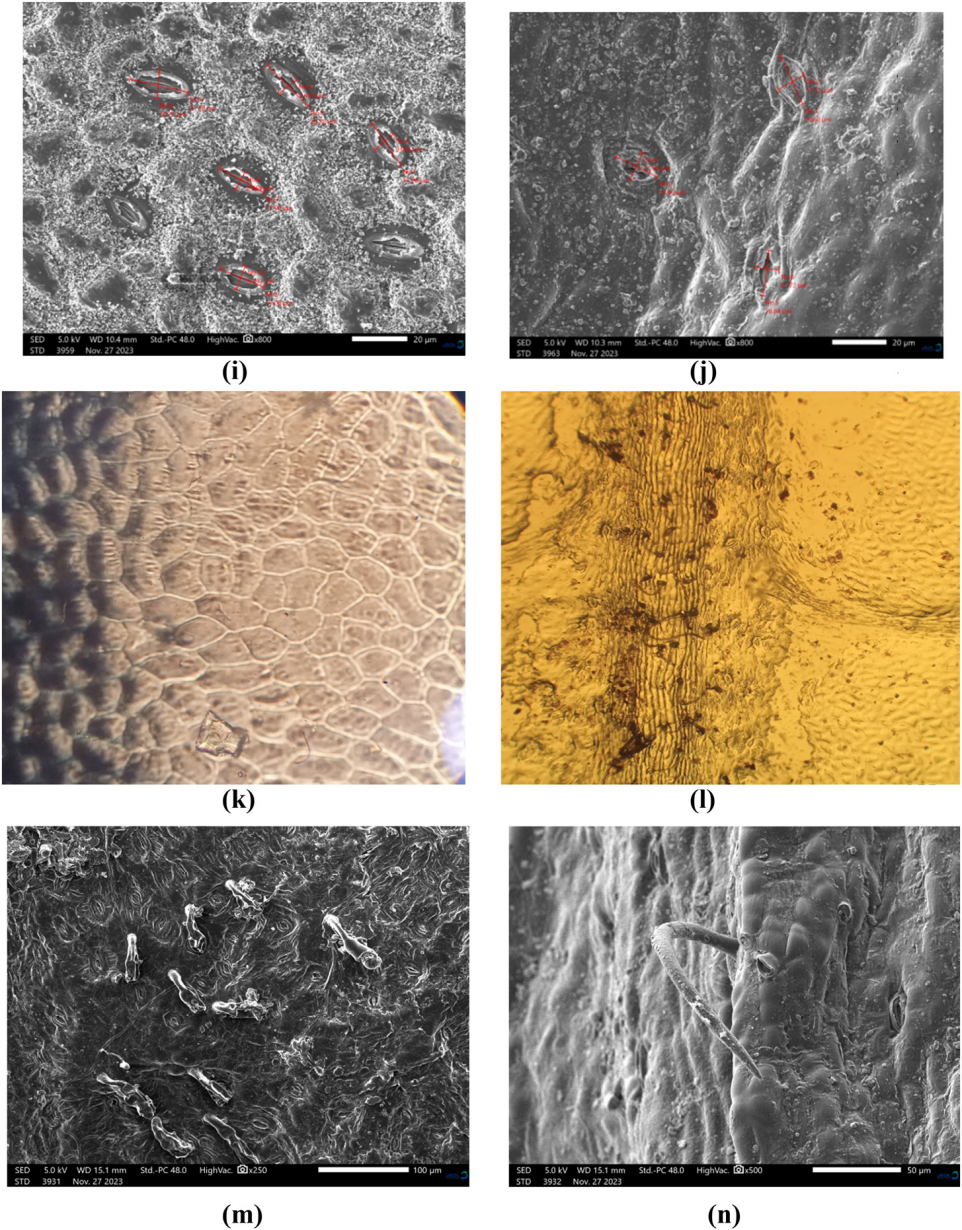
(a) Stomata on the abaxial surface of PA; (b) stomata of the abaxial surface of PT; (c) micrographs of stomata on the abaxial surface of PA; (d) micrographs of stomata on the adaxial surface of PA; (e) size of stomata on the abaxial surface of PA; (f) size of stomata on the adaxial surface of PA; (g) micrographs of stomata on the abaxial surface of PT; (h) Micrographs of stomata of the adaxial surface of PT; (i) size of stomata on the abaxial surface of PT. (j) size of stomata on the adaxial surface of PT. (k) form of the epidermis of PA; (l) main leaf veins of PA; and (m) and (n): ciliated and glandular trichomes of PA.

Three distinct categories of stomata have been identified in all examined leaves, including anomocytic, paracytic, and actinocytic stomata. More than 90% of the leaves exhibited anomocytic stomata, and sometimes a single epidermis had two or more types of stomata (Figure 10). Additionally, in both species, stomata are primarily located on the abaxial surface of the leaves, although they are occasionally present on both surfaces (Table 9).

## 11 Discussion

### 11.1 Dendrometric characteristics

In this study, most dendrometric parameters of the pistachio tree are not influenced by different zones, and the same observations were made concerning acacia and were endorsed by studies conducted by Fonton et al. [28]. However, statistical analysis revealed two discriminant features for identifying PA and PT. First, the canopy of PA covers a larger area than that of PT in the three study zones. El Zerey-Belaskri et al. [29] showed that PA trees are characterized by dense foliage compared to other species of the *Pistacia*, confirming our findings regarding the canopy area. Second, the PT tree has a multi-trunk arboreal stem starting at less than 50 cm from the ground. On the other hand, the PA tree has a well-individualized stem, whether for young shoots or older subjects. This observation was confirmed by Quezel and Santa [30]. The trunk of the PA tree is primarily singular, although in rare cases, it may branch out from the base. On the other hand, similar studies conducted in the literature [2,30] showed that PA trees are robust and vigorous, reaching heights of 15–20 m; however, intermediate trees are also observed. The trunks of adult trees can exceed 1 m in diameter and 6 ms in circumference, with a striated dark gray bark.

Our dendrometric results are partially inconsistent with those of Aloui [31]. He showed that the climatic effect is significant on the tree circumference and canopy diameter but is not a significant factor in tree height.

Regarding the sex ratio, our results align with the study of Mabberley [32]. Flowers are generally unisexual in Anacardiaceae, and the genus *Pistacia* is distinguished by its dioecious reproductive system. Some exceptional cases of sexual types have been reported in the literature; Ozbek and Ayfer [33] observed two hermaphrodite trees in Turkey.

### 11.2 Morphological traits of leaves, inflorescence, and fruits

Among the distinctive features, several are used in the classification of species in the *Pistacia* genus, including the size of the terminal leaflet, the number of pairs of leaflets, the shape of the apex of the terminal leaflet, the presence of leaflet wings on the rachis, and finally, the shape of the fruit [5,8].

The description provided in previous studies [5,8,13,34] suggests that the leaves of *P. atlantica* possess leaflets with finely winged rachis [35]. The rachis is broadly winged, with wings extending along its entire length [13]. Regarding the terminal leaflet, it is stated to be as large as the basal leaflets [34]. In our study, the size of the terminal leaflet varies, and the shape of the apex can be acute or acuminate, contrary to the obtuse shape reported by Zohary [5].

Regarding our observations on inflorescences, the parameters we studied did not uncover any distinctive characteristics. However, during the sampling process, we noticed that some male PA produced flower buds starting in January. Based on our sample set and the studies conducted by Yaaqobi et al. [36] on the flowering and germination of PA seeds, we considered these cases to be exceptional occurrences. On the other hand, flowering is early in trees PA and late in PT and this phenomenon is more pronounced in areas where aridity decreases. It is also crucial to emphasize that flowering occurs before vegetative development, with a tendency for males to flower before females. This observation aligns with Delph [37], demonstrating that males invest more resources in flowering early in the season before leaf production. Thus, resources allocated to the development of vegetative growth, including photosynthetic tissues (leaves), may be limited or unstable [38].

On the other hand, in the mountains of the Bulgarian Rhodopes, some monoecious plants of PT were found [39]. Some trees were entirely monoecious, while others had only staminate flowers or bore only pistillate inflorescences. To assess the stability of the monoecious type, a study was conducted to investigate the morpho-anatomical development of floral buds and the floral biology of the aforementioned genotypes of PT. The study of monoecious plants of PT in the Mediterranean region is important for the opportunity it provides to increase the biodiversity of agroforestry systems, for rootstock platforms, and for obtaining new interspecific hybrids as potential rootstocks [40].

The statistical analyses indicate significant variations both in terms of size and shape of the seeds between the two species in the three stations studied. This observation has been validated within the subspecies of the PA. Indeed, Behboodi [13] described the leaves and fruits of the PA tree found in Iran. The drupes are slightly broader than long in *P. atlantica* subsp. *mutica*; they are globular, with equal length and width in *P. atlantica* subsp. *cabulica*; and they are depressed globular, measuring 5–8 mm × 8–10 mm in *P. atlantica* subsp. *kurdica*. Previous studies conducted on the fruits partially align with our findings. Indeed, the fruit is a syncarpous drupe, morphologically variable, characterized by a thin and oleaginous exocarp [5,6,29]. It is small in size (≤0.8/0.7 cm) [29], with large and oval drupes that turn from red to dark green when mature (August to September). Its single seed is oval and yellow. Previous results report an average of 37 fruits per infructescence in adult individuals [41].

The measurements taken on the size of the fruits and almonds of PT and PA trees reveal a significant issue of empty seed formation, estimated to be 77%. This result has been explained in previous studies [42,43]; the authors suggest that the production of fruits with non-viable seeds can be triggered by specific conditions affecting a plant during the pollination period or during the process of fertilization and early fruit growth [44].

## 12 Micromorphological characteristics

Stomata represent the primary mechanism by which higher plants adapt to their constantly changing environment [45]. Homeostasis, a fundamental biological property of living organisms, requires the regulation of the internal environment while interacting with the surroundings [46]. Adaptive homeostatic ranges are significantly broader in plants than in animals [47], but the overall conservation of homeostasis remains equally crucial in both kingdoms.

The results obtained from measurements of qualitative and quantitative characteristics of stomata and trichomes agree with some studies but differ from others. Statistical analyses reveal significant differences for the measured variables among the studied species. According to the statistical results, the most discriminating stomatal characteristics recorded during this study are size and stomatal density.

Examining the data, the density is higher in the abaxial epidermis, and it appears that increased aridity is associated with a decrease in the number of stomata. This trend is observed on both the adaxial and abaxial surfaces, and similar results were reported by Gindel [48] in studies conducted in Israel. PT exhibits a higher stomatal density than PA. Additionally, PT develops stomata of smaller size than PA. The few stomata on the adaxial surface are primarily located near the main and secondary veins of the leaflet. These observations support the earlier findings of Doghbage et al. [49].

Our conclusions are inconsistent with the phytodermological study of *Pistacia atlantica* subsp. *atlantica* and *Pistacia lentiscus* conducted by Smail-Saadoun [50]. This study demonstrated that the adaptation of these two species to water scarcity involves a complete absence of stomata on the upper surface of the leaves and the presence of paracytic mesoperigenous stomata on the lower surface of the leaf.

About 90% of the samples exhibit anomocytic forms, and in rare cases, actinocytic and paracytic stomata are observed in both species (Figure 10a and b). Most epidermal cells have an irregular shape, except for 10% of the cells in PT from the Afourgagh area, which have a polygonal shape. Furthermore, the guard cells were not aligned at the same level as the adjacent epidermal cells, and the subsidiary cells did not clearly surround the guard cells. Our results are in agreement with those of Al-Saghir et al. [51] and contradict the findings of the study conducted by Lin et al. [3], which reported that all pistachio species (*P. atlantica, P. exicana, P. chinensis, P. integrrima, P. khinjuk, P. texana, P. vera, and P. weinmannifolia*) had actinocytic stomata.

According to Al-Saghir [52], amphistomy is considered the primitive characteristic in *Pistacia* species, with occurrence on a single surface (hypostomy or epistomy) seen as an evolution. *P. vera* is identified as the most primitive species, while *P. terebinthus* is considered more recent. In our study, PA exhibits almost the same stomatal density on the abaxial surface compared to PT in Afourgagh and Amghas, but it is lower on the adaxial surface, where stomata are localized along the main and secondary veins in all three studied zones. The researchers [10] suggest that stomatal loss in *Pistacia* species could be an adaptation to climate changes. This adaptation is also supported by morphological features, as more evolved species have smaller elongated leaflets with pointed tips, facilitating water drainage from the leaves.

The outcome of Simon [53] considered that the regulation of evaporation at the stomatal level can occur either through their sunken position or by the presence of thick edges that protect the pore [53]. Mucilage deposited on stomatal pores could also contribute to reducing evaporation. Wax deposits would serve a similar function, especially in species with low hair coverage.

The information regarding the presence or absence of trichomes on *Pistacia* leaves is contradictory. Al-Saghir et al. [51] reported the presence of trichomes in all *Pistacia* species, while other authors indicated that *Pistacia* leaves are devoid of hairs [3]. The results of the present study reveal the absence of trichomes on the leaflets of PT. Many immature leaves lose their trichomes as the leaf blade opens and expands [54] showed that trichomes produce essential oils that appear to play a protective role against herbivores and pathogens.

## 13 Conclusions

This study has unveiled crucial aspects for the identification of two species within the *Pistacia* genus, examining dendrometric, macromorphological, and micromorphological characteristics. Parameters such as canopy surface and trunk count have been identified as distinctive indicators between these two species. Additionally, features including leaf and terminal leaflet dimensions, as well as the size and shape of the terminal leaflet, fruit and almond size, stomatal size and density, and the shape of epidermal stomata and trichomes, were recognized as the most discriminating. Given the broad distribution of these species in North Africa, particularly PA subjected to diverse climatic conditions, it is crucial to conserve these resources and utilize them optimally. This holds particular significance within the context of research and development projects, especially those aimed at restoring heavily degraded forest environments in the Middle Atlas region of Morocco. Furthermore, the potential presence of genetic variability underscores the need to delve deeper into research in this area, considering the comparison of populations from different geographical regions through the use of molecular markers.
